# A comparison of ketamine-midazolam combination and propofol-fentanyl combination on procedure comfort and recovery process in pediatric colonoscopy procedures

**DOI:** 10.12669/pjms.37.2.2787

**Published:** 2021

**Authors:** Sedat Saylan, Ulas Emre Akbulut

**Affiliations:** 1 Sedat Saylan, MD. Faculty of Medicine, Department of Anesthesiology, Karadeniz Technical University, Trabzon, Turkey; 2 Ulas Emre Akbulut, Department of Pediatric Gastroenterology Hepatology & Nutrition, University of Health Sciences, Antalya Education and Research Hospital, Antalya, Turkey

**Keywords:** Colonoscopy, Child, Sedation, Ketamine, Propofol

## Abstract

**Objective:**

To compare the efficacy and safety of midazolam-ketamine combination versus fentanyl-propofol combination in pediatric diagnostic colonoscopy.

**Methods:**

This is a retrospective study of 68 children undergoing diagnostic gastroenterology with midazolam-ketamine combination (Group-K) or with fentanyl-propofol combination (Group-P) in the pediatric gastroentology department at a Turkish tertiary hospital between January 2015 and June 2017. An intravenous midazolam was administered one minute before ketamine administration in Group K. Intravenous fentanyl was given to Group P, followed by intravenous propofol.

**Results:**

There were statistically no significant differences between the groups as for age, gender, weight, duration of colonoscopy and complications observed during procedure. Ramsay sedation score was significantly higher in Group K. Recovery time and the rate of complications during the recovery of Group-K (23 patients, 65.7%) was significantly higher than that of Group P (8 patients, 24.2%) (p= 0.001).

**Conclusions:**

Colonoscopy procedures can be quite comfortable in children when using the midazolam-ketamine combination. However, adverse effects related to ketamine were observed during recovery.

## INTRODUCTION

Colonoscopy is a painful procedure due to the mesenteric traction maneuvers and colonic distension by gas insufflation and the device frequent winding inside the intestine.^1^,^2^ Adult patients endure colonoscopy with conscious sedation; however, pediatric patients frequently need deep sedation or general anesthesia due to their relatively high level of anxiety, lack of cooperation, and pain perception.^3^

Ketamine has been one of the most commonly used agents for sedation and analgesia in children undergoing outpatient’s procedures.^4^-^6^ However, side effects such as aspiration, stridor, laryngospasm and after-sedation nausea, delirium and physical aggression have been reported.^4^ Ketamine is used in combination with benzodiazepines to reduce the frequency of these side effects.^5^,^7^ A sedative-hypnotic drug, propofol offers a fast start and a short rehabilitation time and readily enables a convenient level of sedation without having any analgesic properties.^8^-^10^ However, its high doses can cause hypotension and respiratory depression.^11^,^12^ Fentanyl is used in combination with propofol as an adjuvant to allow for effective use of propofol at lower doses.^13^

This study aimed to make a comparison between the sedative efficacy and safety of midazolam-ketamine combination and fentanyl-propofol combination in children having colonoscopy and to find out the most convenient sedation method.

## METHODS

We reviewed retrospectively the drug combinations midazolam-ketamine (Group K) and fentanyl-propofol (Group P) used in our clinic for the sedoanalgesia of pediatric patients who underwent diagnostic colonoscopy in the pediatric gastroenterology department at a tertiary hospital between January 2015 and June 2017. Ethical permission to conduct this study was obtained from the Local Ethics Committee (Ref. No: 2018/35, dated 05/06/2018). The study was conducted in compliance with the Declaration of Helsinki. Demographic characteristics of all patients, vital signs, duration of induction, number of drugs used, cecum intubation, sedation time, recovery time and observed complications were recorded.

Sedation was not administered to children with respiratory tract infections, glaucoma, psychosis, hypertension, porphyria, metabolic or neurologic diseases, increased intracranial pressure and intracranial mass or if the sedation type was not approved by the anesthesiologist. In addition, according to American Association of Anesthesiologists, patients with ASA 3 and above and patients undergoing therapeutic colonoscopy were excluded from the study.^14^

During the procedure, heart rate, oxygen saturation, noninvasive arterial blood pressure and modified Ramsay Sedation Score (RSS) of all patients were regularly monitored and recorded by the anesthesiologist.^15^

All patients were given oxygen (2 L/minutes) by nasal cannula starting from anesthesia induction until the end of the procedure. Complications such as apnea, laryngospasm and cardiac arrest during the procedure were defined as major complications, while arrhythmia, desaturation, bradycardia, increased oral secretions, hypotension, tachycardia, coughing, flushing, and vomiting as minor complications. According to RSS, patients’ responses to stimuli were scored between one and six. Scores ≥5 indicate adequate sedation allowing for invasive intervention. An intravenous 0.1 mg/kg (maximum 4 mg) bolus dose of midazolam was conducted three minutes before ketamine administration in Group K. The verbal and tactile stimuli were administered and the patients’ responses to them were evaluated following 1 mg/kg bolus dose ketamine.

If sufficient degree of sedation was not achieved, 0.5 mg / kg additional ketamine was applied. The procedure was started after sufficient sedation and upon no patient response. Intravenous fentanyl bolus dose-1 μg/kg was administered to Group-P. Three minutes later, one mg/kg bolus dose propofol was administered intravenously. The patients’ responses were evaluated with verbal and tactile stimuli one minute after propofol administration. Propofol 0.5 mg/kg was added at one-minute intervals if adequate sedation was not achieved. The procedure was started upon sufficient sedation and no response.

Modified Aldrete scores were used to assess recovery.^16^ Endoscopy unit discharge was allowed upon ≥ 9 Aldrete scores. Any complications such as double vision, agitation, dizziness, hallucinations, and nausea during recovery were recorded.

The power analysis was performed as posthoc in the sample size study. The power was obtained 81.1% when effect size was considered as Cohen d=0.7, with type-1 error 0.05, 35 in Group K and 33 in Group P (G. Power version 3. 9.1.2, Germany).

Statistical Package for Social Sciences (SPSS Inc., Chicago, IL) Version 23.0 was applied for the statistical analysis. Mean±standard deviation (SD) and Median (range) were indicated as descriptive data. The independent paired samples t-test was used to compare normally distributed variables between groups, while the Mann-Whitney U test was used for non-normally distributed variables. The categorical variables were compared using Chi Square test. P-values <0.05 were recognized as statistically significant.

## RESULTS

**Fig.1:**
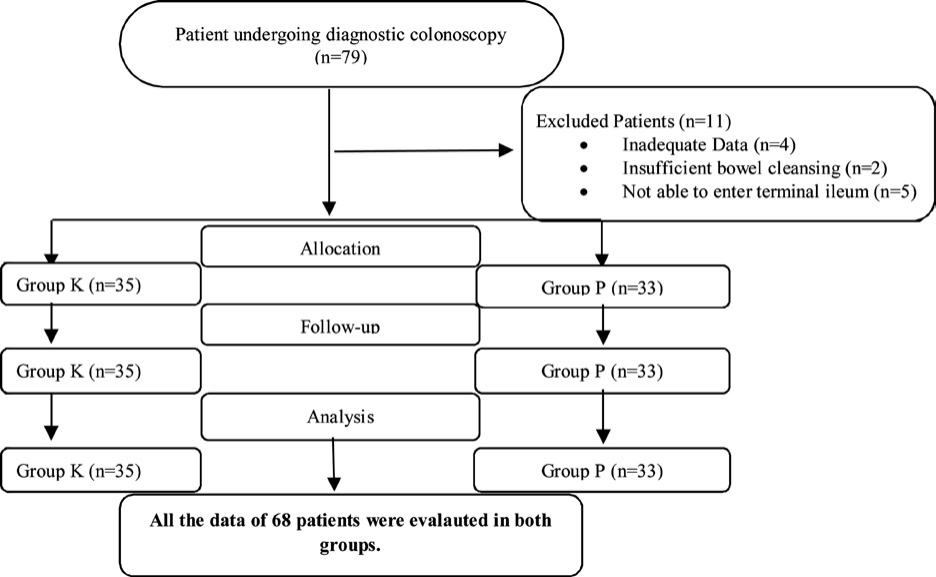
Flow chart of the study.

Sixty-eight diagnostic colonoscopy procedures with a midazolam-ketamine combination or with fentanyl-propofol combination sedation were performed in the course of study. Four patients with inadequate data, two patients with poor bowel preparation and five patients who could not enter the terminal ileum were excluded from the study (Fig.1).

Sixty-eight patients (M age: 10; range: 4–17 years; mean SD: 9.97 ± 3.09 years; M weight: 35 kg; range: 15–87 kg; mean ± SD: 35.27 ± 15.94 kg) were in final groups. There were no significant differences between the groups regarding colonoscopic indications (Table-I).

**Table-I:** Patient characteristics.

*Variable*	*Group K (n=35)*	*Group P (n=33)*	*p value*
Age, years, mean ± SD	10.05 ± 3.78	10.34 ± 3.90	0.853
Male, n (%)	17 (48.6)	14 (42.4)	0.610
Weight, kg, mean ± SD	31.20 ± 16.55	35.26 ± 15.96	0.651
***Indications of colonoscopy, n (%)***			
Rectal bleeding	12 (34.2)	13 (39.4)	0.662
Chronic abdominal pain	11 (31.4)	10 (30.3)	0.920
Chronic diarrhea	10 (28.6)	9 (27.2)	0.905
Anemia	2 (5.7)	1 (3.0)	N.A.

Induction time, cecum intubation time, sedation time, recovery time, sedative drugs dosages and RSS in both groups are presented in Table-II. Significant differences were not detected between groups as for induction time, cecum intubation time and sedation time. Intra-operation Ramsay sedation score and the recovery time were essentially higher in Group K.

**Table-II:** Evaluation of patients during and after endoscopy.

*Variable*	*Group K (n=35)*	*Group P (n=33)*	*p value*
Induction time, mean ±SD (range), min	1.40 ± 0.40 (1-3)	1.45 ± 0.50 (1-3)	0.547
Cecum intubation, mean ±SD (range), min	6.72 ± 1.20 (4-10)	7.10 ± 1.46 (4-11)	0.195
Sedation time, mean ±SD (range), min	13.38 ± 3.29 (9-18)	14.76 ± 4.56 (9-19)	0.258
The recovery time, mean ± SD (range), min	48.15 ± 20.37 (15-110)	32.57 ± 14.85 (10-80)	*0.006*
Average sedative dose, mean ±SD (range), mg/kg	1.55 ± 0.36 (1.0-2.0)^a^	1.63 ± 0.49 (1.2-2.1)^b^	
***Ramsey Sedation Score, n (%)***			
Score 4	5 (14.3)	13 (39.4)	0.021
Score 5	10 (28.6)	11 (33.3)	
Score 6	20 (57.1)	9 (27.3)	

a ketamine

b propofol

Patients did not develop any major complications such as apnea, cardiac arrest, or laryngospasm but six patients developed minor complications (17.1%) in Group K and four (15.1%) in Group P (p=0.558). Three patients (8.6%) in Group K had increased oral secretion which constitutes a risk for aspiration and laryngospasm; however, none of the patients experienced them. None of our patients terminated the procedure due to insufficient sedation or any complications. A total of 31 patients (45.5%) developed complications during the recovery, the most frequent of which was dizziness (17 patients, 25.0%). Complication rates of Group K (23 patients, 65.7%) were a lot higher than those of Group P (8 patients, 24.2%) (p< 0.001) (Table-III). Two patients (5.7%) developed emergence reactions in Group K but none in Group P.

**Table-III:** Complications in the two groups.

*Variable*	*Group K (n=35)*	*Group P (n=33)*	*p value*
Complications during the procedure, n (%)	6 (17.1)	4 (15.1)	0.558
Tachycardia, n (%)	1 (2.8)	1 (3.0)	N.A.
Bradycardia, n (%)	0 (0.0)	1 (3.0)	N.A.
Increased oral secretions, n (%)	3 (8.6)	0 (0.0)	N.A.
Cough, n (%)	2 (5.7)	2 (6.0)	0.951
Complications during the recovery, n (%)	23 (65.7)	8 (24.2)	***<0.001***
Agitation, n (%)	7 (20.0)	0 (0.0)	N.A.
Emergence reaction, n (%)	2 (5.7)	0 (0.0)	N.A.
Hallucinations, n (%)	4 (11.4)	0 (0.0)	N.A.
Double vision, n (%)	15 (42.8)	2 (6.0)	***<0.001***
Dizziness, n (%)	13 (37.1)	4 (12.1)	0.017
Nausea, n (%)	3 (8.6)	2 (6.0)	0.691

## DISCUSSION

This retrospective study compared the efficacy and safety of midazolam- ketamine combination versus fentanyl-propofol combination in pediatric diagnostic colonoscopy. This study confirms that both midazolam-ketamine and fentanyl-propofol combinations provide effective sedation in pediatric diagnostic colonoscopy. However, the procedures performed with the midazolam–ketamine combination were more comfortable than the fentanyl–propofol group and the fentanyl–propofol group was more comfortable in the recovery period in terms of complications.

Ketamine is a dissociative anesthetic characterized by potent analgesia, sedation, and amnesia while protecting spontaneous ventilation.^4^ It also has a protective sympathomimetic activity preserving heart rate and blood pressure.^17^ 1.5-2.0 mg/kg loading doses of ketamine are commonly proposed to provide dissociative state. However, its increased doses might cause prolonged recovery period, emergence delirium, hallucination, visual problems, nausea, vomiting and laryngospasm.^4^,^18^,^19^ Previous studies have showed that ketamine-midazolam combination is more effective and has fewer side effects than ketamine alone.^5^,^6^ Propofol-based sedation is reported to be a safe and efficient option for pediatric gastrointestinal endoscopic procedures.^8^,^19^ However, propofol has some disadvantages as well such as diminishing cardiac contractility, systemic vascular resistance, and cardiac outflow and causes respiratory depression in high doses.^4^,^11^,^19^ Another cause for concern in terms of propofol use is the absence of an antidote to cancel these adverse effects.^21^ Propofol in combination with midazolam or fentanyl was shown to provide greater comfort during the procedure leading to fewer side effects.^11^,^22^

In our previous prospective study in children who had undergone upper gastrointestinal endoscopy (UGE), the success rate was 100% with 0.1 mg / kg midazolam and 1.03 mg / kg ketamine in average and 99.2% with 1 μg/kg fentanyl and 1.46 mg/kg propofol in average.^4^ In this study, we found 100% success rate in both groups. However, the average dose of ketamine and propofol was 1.50 mg / kg and 1.64 mg / kg, respectively. Colonoscopy is a more painful procedure than UGE, which may explain the need for more drug doses. No major complications were seen in any patients although drugs were used at higher doses. Observed minor complications did not affect the success and comfort of the procedure. Although complete success was achieved in both groups, the comfort during the procedure was found to be better in the midazolam-ketamine group. However, early recovery and complications during awakening are important parameters in daily procedures requiring sedation such as colonoscopy. In our study, it was observed that the duration of awakening and complications were more frequent in the midazolam-ketamine group compared to the fentanyl-propofol group. Agitation and emergence reactions during sedation with ketamine are important side effects. Particularly, emergence reactions cause unrest in families.

Canbolat et al. compared the combination of ketamine-propofol (KP) and ketamine-dexmedetomidine (KD) as a sedoanalgesia method during tooth extraction in children.^23^ There was no statistically significant difference between the groups in terms of preoperative and postoperative anxiety scores or postoperative first- and second-hour pain scores. Although the KP and KD combinations provide effective deep sedation for tooth extraction for non-cooperative children with severe anxiety, they emphasized that the KP combination provides better surgeon satisfaction levels and causes less nausea and vomiting. Therefore, they stated that KP could be a better option for tooth extraction in children. In our study, Ramsay sedation score was higher in the combination of ketamine-midazolam, thus providing more ease of operation.

Arpaci et al. also compared ketamine and inhaler anesthesia for sedoanalgesia during tooth extraction in children.^24^ The authors emphasized that ketamine is an amnestic, analgesic, hypnotic effective and safe agent, without altering pharyngeal and laryngeal reflexes, minimizing the possibility of aspiration during the procedure. They stated that ketamine may be preferred over other agents for pediatric sedoanalgesia. They also found that postoperative agitation was higher in children under inhaler anesthesia compared to ketamine. In our study, although an increase in oral secretionwas detected in three patients (8.6%) in Group K, which creates a risk of aspiration and laryngospasm, none of the abovementioned complications occurred.

The incidence of emergence reactions increases especially when ketamine is used at high doses, when a fast injection (< 1 minute) is administered and when excessive visual or verbal stimuli exist during the recovery.^25^ In the present study, we used 1.50 mg/kg ketamine and the emergence reaction was 5.7% and agitation 20%. None of the patients with the propofol-fentanyl combination developed emergence reactions or agitation.

### Limitations to the study

Firsty, this is a retrospective study. Secondly, it considered only diagnostic colonoscopy procedures. In addition, therapeutic procedures may require different drug dosages. This study did not include patients below age four which made it impossible to examine likely problems within younger patients, and notably in infants. Lastly, such factors as the nature of the endoscopist or nurse training may have influenced this single-centered study, which can be clarified with further multi-centered studies.

## CONCLUSION

Midazolam-ketamine and fentanyl-propofol combinations provide adequate sedation for children during colonoscopy. In none of our patients we had to terminate the procedure due to insufficient sedation or any complications. Although there were temporary complications such as double vision and dizziness in the ketamine-midazolam group during the recovery period, the intraoperative RSS was higher in the ketamine-midazolam group. Therefore, we consider that pediatric colonoscopy operations could be truly satisfying with the ketamine-midazolam combination. Additional studies are required to determine the competence of this sedation procedure in younger patients and in curative procedures.

### Author’s Contribution:

**SS:** Conceived and designed the study and is responsible and accountable for the accuracy and integrity of the work.

**SS & UEA:** Did data collection, statistical analysis, manuscript writing, review and final approval of manuscript.
